# Case report: Zone 3 gunshot wound to the left neck treated with combined Foley catheter balloon tamponade and interventional radiology stent placement

**DOI:** 10.1016/j.tcr.2025.101159

**Published:** 2025-04-22

**Authors:** Rebekah L. Bjorklund, Luis Fernandez, Gary Spiegel, Brittany Wagner, Austin Eagleton

**Affiliations:** a University of Texas Health Science Center at Tyler, TX, United States of America; b Dept. of Surgery, Div. of Trauma Surgery/Surgical Critical Care, The University of Texas Health Science Center, Tyler, TX, United States of America; c The University of Texas-Tyler School of Medicine Inaugural Bill Barrett Endowed Chair in Trauma Surgery, Director, Trauma Wound Care, UT Health East, Tyler, TX, United States of America; d Associate Professor of Surgery Div. of Trauma Surgery/Surgical Critical Care, The University of Texas Health Science Center, Director Trauma ICU, UT Health East, Tyler, TX, United States of America; e Department of Interventional Radiology, The University of Texas Health Science Center, Tyler, TX, United States of America

**Keywords:** Trauma, Critical care, Zone 3, Neck, Interventional radiology, Carotid stent

## Abstract

In the trauma and military setting, catheter balloon tamponade has been successfully used as an effective tool for the management of exsanguination secondary to penetrating injuries. This maneuver may improve outcomes in patients with uncontrolled bleeding, particularly in penetrating neck injuries (PNI), thorax, axillae, and groin. We describe a case of a stab wound to the left neck that led to surgical left neck exploration utilizing Foley catheter balloon tamponade (FCBT) for initial control of massive hemorrhage and a subsequent interventional radiology (IR) endovascular stent placement for a zone 3 internal carotid artery injury.

## Introduction

Balloon catheter tamponade (BCT) has been used to temporarily control massive hemorrhage in many surgical settings [1]. Initially described for esophageal varices [2], it has been effectively applied to patients with traumatic vascular and solid organ injuries [3]. BCT and the FCBT variant have been successfully deployed to control many forms of massive bleeding, such as facial, cardiothoracic, gastrointestinal, solid organ, peripheral vascular, postpartum hemorrhage, and, as in our case, carotid artery injuries [4]. Gilroy et al. at Baragwanath Hospital in Johannesburg, South Africa, were the first to describe using FCBT to control significant hemorrhage following PNI [5]. FCBT has been effective in the control of hemorrhage in the field as well as in the hospital setting [6].

In most cases, operative treatment for PNI remains the mainstay for the unstable trauma patient, while non-operative management and endovascular management are usually reserved for the stable patient [7]. We present a case that required FCBT intra-operatively for control of hemorrhage for a zone 3 internal carotid artery injury and subsequent IR endovascular stent placement.

## Case report

A 30-year-old male with no significant past medical history presented as a level 1 trauma transfer from an outside hospital (OSH) with a presumed gunshot wound to his left neck. The patient underwent intubation for agonal breathing and had a left neck wound with significant bleeding and reported GCS 6. A triple lumen right internal jugular central line was placed at that time. The patient received four units of pack red blood cells, Tranexamic acid (TXA) and was transferred to our facility.

The patient arrived intubated, tachycardic, and hypotensive with clinical signs of hemorrhagic shock. Upon removal of the pressure dressing from the left neck, bright red, arterial, pulsatile bleeding was noted. A massive transfusion protocol (MTP) was activated. A chest x-ray and neck x-ray were obtained to assess bullet trajectory, and the patient was brought to the operating room within 15 min of arrival at the trauma bay. The patient was rapidly prepped and draped in a sterile fashion, and bleeding was controlled with direct pressure on the wound.

**Fig. 1:**
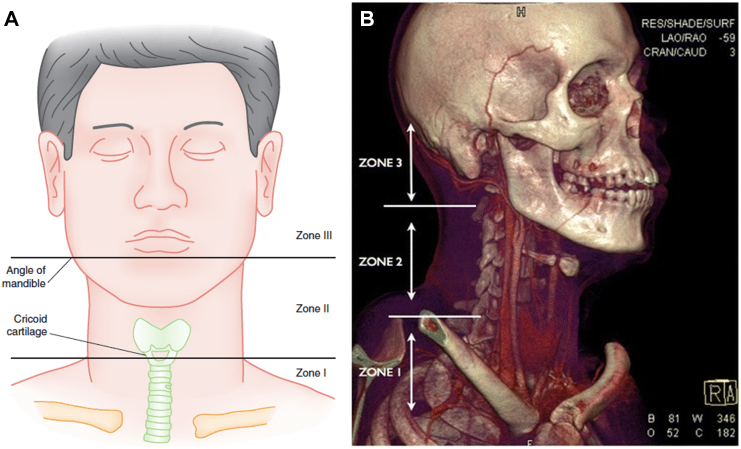
Zones of the neck: frontal (A), right lateral (B) orientation.

A standard left-neck incision was made along the anterior border of the sternocleidomastoid muscle. There was significant right medial displacement of all structures due to a massive hematoma. Direct pressure continued to be held, and an intra-operative sterile Doppler probe was used to assist in the dissection of the common carotid artery (CCA). Proximal control of the CCA was obtained with a DeBakey clamp and Rummel tourniquet with umbilical tape, and pressure was released from the proximal internal carotid artery (PICA). Rapid, significant bleeding obscured the field due to back bleeding from the circle of Willis. Attempts were made to extend the dissection cephalad. However, it became obvious that the injury initially reported as a zone 2 injury was a zone 3 neck injury (Fig. 1: A, B) [8,9].

**Fig. 2:**
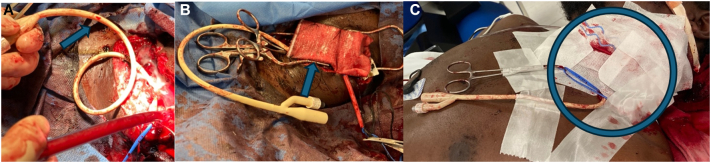
A: Foley balloon (arrow) deployed along the injury tract into Zone 3. B: Kaolin-based dressing (arrow) was applied without further bleeding. C: Temporary dressing applied for transport to the interventional radiology suite (circle).

The backup trauma surgeon was called and inserted a 12 French Foley catheter along the injury tract into Zone 3. The balloon was filled with sterile saline, and the back bleeding from the internal carotid artery was controlled. A military, kaolin-based hemostatic bandage (QuikClot® Teleflex INC., Wayne, Pennsylvania, USA) was applied with direct pressure, and no further bleeding was noted (Fig. 2: A-C). The left neck incision remained open, the foley was sutured, and the Rummel and DeBakey clamp remained in position while the patient was transferred to interventional radiology.

A portable digital radiograph was obtained to assess for a possible bullet trajectory; however, given no exit wound and no bullet visualized, this suggested that the injury was a Zone 2 to 3 stab wound instead of a gunshot wound. Neurointerventional radiology (NIR) was consulted for coil embolization of the internal carotid artery (ICA) versus endovascular stent placement, and the patient was transported to the interventional radiology suite.

**Fig. 3:**
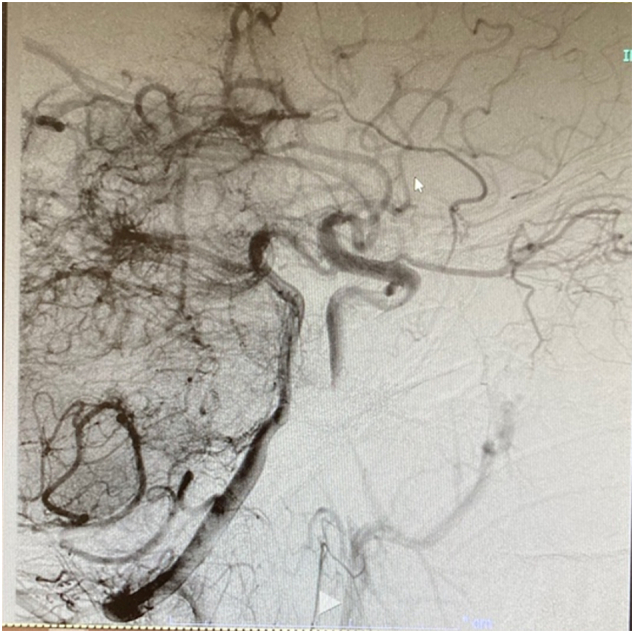
IR access was obtained from the groin, and a cervical-cerebral angiogram revealed normal runoff except for the left common carotid artery secondary to the DeBakey clamp that remained in place.

IR access was obtained from the groin, and a cervical-cerebral angiogram revealed normal runoff except for the left common carotid artery secondary to the DeBakey clamp that remained in place. There was rapid filling of the left anterior cerebral and middle cerebral artery with slow retrograde filling of the cavernous segment of the left internal carotid artery. There was limited filling of the posterior frontal and parietal branches. However, no occlusions were evident (Fig. 3).

**Fig. 4:**
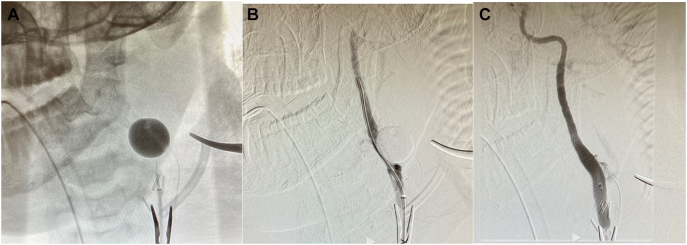
A: Foley balloon reinflated with contrast. B: Lesion crossed with 035 guidewire. C: Viabahn Endograft stent (9 mm x 60 mm) deployed.

At this time, the decision was made to attempt to cross the lesion (C2 ICA) antegrade instead of retrograde from the right internal carotid artery. The proximal common carotid artery clamp was released, and an angiogram was obtained, revealing minimal filling of some of the proximal external carotid branches and possible filling of the proximal portion of the left internal carotid artery that was not well demonstrated. A BENCHMARK™ BMX®96, Access System catheter was advanced beyond the proximal clamp, and the foley balloon was deflated to allow a repeat angiogram of the left internal carotid artery, revealing significant retrograde bleeding. The foley balloon was reinflated with contrast medium to assist with the trajectory of the internal carotid artery (Fig. 4, A).

Using a 0.035 guidewire, the lesion was crossed and confirmed with an angiogram (Fig. 4, B). Given that adequate control was obtained, it allowed time to exchange and place a 9 mm × 60 mm GORE® VIABAHN® Endoprosthesis vascular stent (Fig. 4, C).

The foley balloon was deflated, and the DeBakey clamp was removed with minimal oozing from the field. A completion angiogram was obtained, which revealed minimal extravasation. There was still diminished flow through the frontoparietal branches of the left middle cerebral artery (MCA). The left neck skin incision was closed loosely, and the FCBT remained slightly inflated but sutured in place to provide gentle external compression, as was the Rummel tourniquet placed around the CCA. The patient remained intubated and was brought to the intensive care unit post-operatively.

**Fig. 5:**
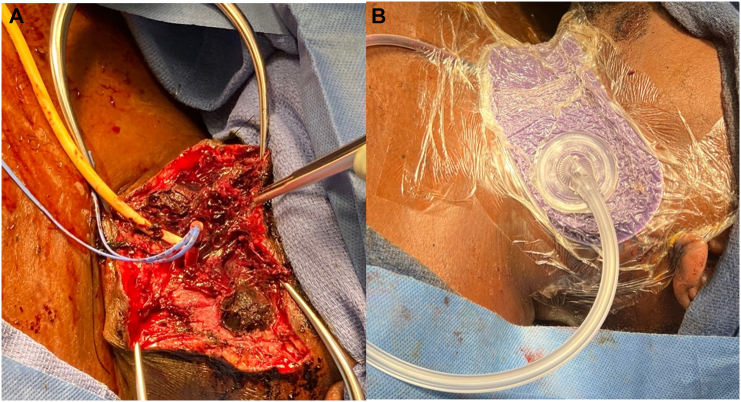
A: Foley catheter just prior to removal. B: The incision was closed, and a ciNPWT dressing was applied.

The patient returned to the operating room three days later for a second look procedure. The FCBT was completely deflated, and there was no active bleeding. It was removed from the surgical field, as well as the Rummel tourniquet. The wound was irrigated with HOCL (Vashe®. Urgo Medical North America, 00 Lexington St Suite 400, Fort Worth, TX, USA). A Jackson-Pratt 7 Fr drain was placed, and the incision was closed with interrupted 3O Vicryl sutures for the platysma and interrupted 3O nylon sutures for the skin. A 13 cm closed incision negative pressure (ciNPWT) dressing was applied (3M™ Prevena™ 125 Therapy Unit, Saint Paul, MN, USA [Fig. 5A, B]).

**Fig. 6:**
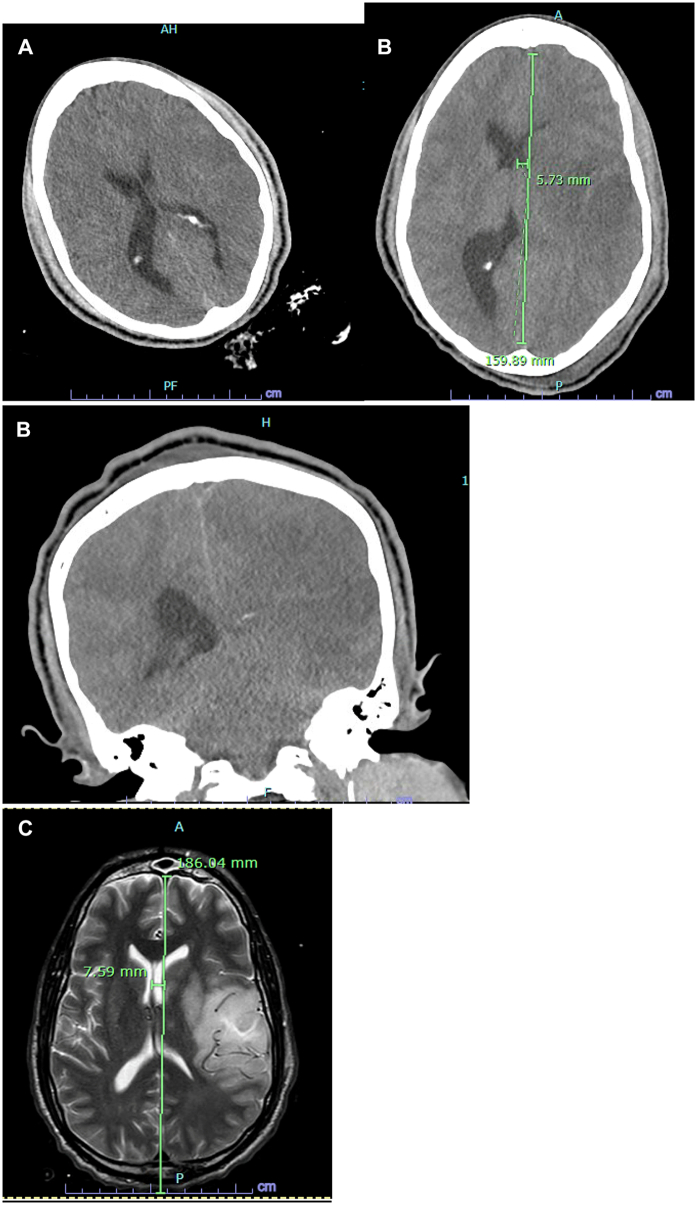
A: CT Head on the hospital/post-operative day 0 concerning loss of gray-white matter differentiation involving the left temporal lobe concerning the left middle cerebral artery (MCA) infarct. B: CT Head on hospital day 3 showing increasing midline shift and increasing ventricular dilation of the right lateral ventricle with large evolving left MCA infarct. C: MRI on hospital day 5 showing large left MCA acute infarct with edema and 8 mm of left to right midline shift

The patient remained intubated due to cerebral edema with a large evolving left MCA infarct which would possibly be embolic as filling defects noted at time of initial IR procedure (Fig. 6, A-C)—the Neurosurgery service placed an external ventriculostomy. The patient was treated for the MCA infarct per current guidelines and continued to improve neurologically. No further imaging to assess the flow through the left ICA was obtained. Before extubation, otolaryngology was consulted to evaluate for a possible recurrent laryngeal nerve injury. He underwent a laryngoscopy evaluation, which revealed complete left vocal cord paralysis. He was ultimately extubated 15 days later and discharged 25 days after the initial presentation to neuro rehab with aspirin and Plavix (due to the existing carotid stent) with a Glasgow Coma Scale score of 14 and limited right lower extremity weakness and right upper extremity paralysis.

## Discussion

Carotid artery injury from PNI remains rare in the literature, and injuries that appear to traverse both zone 2 and involve zone 3 are rarer still. Kong et al. performed the largest retrospective study to date on FCBT in PNI and only had 2 carotid artery injuries (out of 1581 PNI) [17] and Navsaria reports only 1 carotid artery injury (out of 220 PNI) [18]. Open operations for this type of injury are reserved for unstable patients with “hard” signs of vascular injury [10]. Stable patients can undergo further imaging to determine the exact injury and undergo appropriate treatment afterward, whether non-operative, operative, or endovascular treatment [11,12].

Madsen et al. suggested a no-zone approach to initial neck injuries. They found that approximately 40 % of the injuries they looked at either were not correlated to the external location or could not be determine [13]. This was the case with our patient. However, it was obvious that our patient had suffered a carotid injury; it was not clear that the level of the carotid injury would traverse Zone 2, and the laceration to the ICA would be in Zone 3. Given the improvement in high-resolution CT imaging, many are moving away from the traditional algorithm based on neck zones [19].

**Fig. 7:**
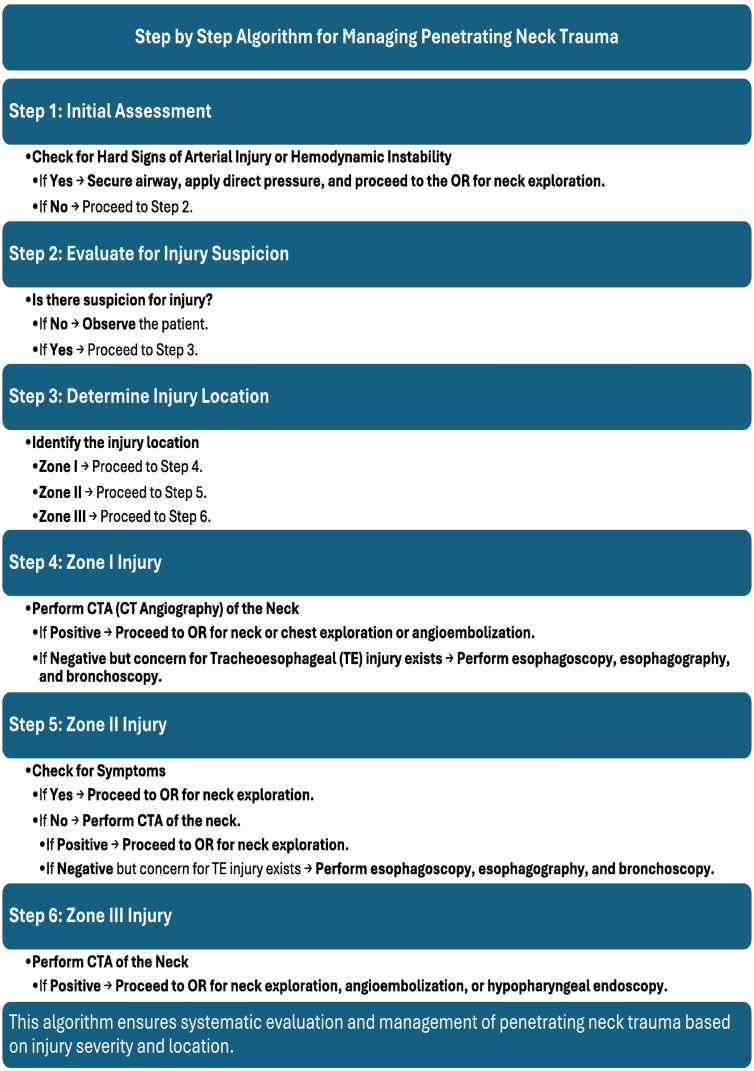
Algorithm for the management of penetrating neck injuries. Hard signs include active bleeding, expanding neck hematoma, air bubbling from the wound, neurologic deficit, and hematemesis. *CTA*, Computed tomography angiography; *OR*, operating room; *TE*, tracheoesophageal. (Modified from Sperry JL, Moore EE, Coimbra R, et al.: Western Trauma Association critical decisions in trauma: Penetrating neck trauma. *J Trauma Acute Care Surg* 75:936–940, 2013.) SOURCE: https://www.surgicalcore.org/popup/417026.

Blitzer et al. reviewed the National Trauma Bank, looking at penetrating carotid injuries from 2002 to 2016 [14]. Their analysis showed no significant difference in patient outcomes for those who underwent open versus endovascular repair. Overall, immediate, emergent operative intervention is reserved for patients with “hard” signs of vascular injury (Table 1) [7,15]. Sperry JL, Moore EE, Coimbra R, et al., suggests stable patients should complete their trauma workup with further vascular contrast imaging and possible IR interventional techniques (Fig. 7) [15].

**Table 1:** Hard signs indicating immediate surgical exploration in penetrating neck injury.

Pulsatile bleeding or expanding haematoma	Audible bruit or palpable thrill	Airway compromise
Wound bubbling	Subcutaneous emphysema	Stridor
Hoarseness	Difficulty or pain when swallowing secretions	Neurological deficits

## Limitations

This manuscript suffers from the common limitations of a case report, (i.e. inability to generalize the data presented, the inability to establish a cause-effect relationship, among others). The advantage of case reporting is its ability to detect novel findings, and it remains the only way to present subject matter that is unusual, rare, or has never been observed previously that might be of importance to the medical reader [16].

## Conclusion

The patient presented in this report was in extremis, with active hemorrhage from a high internal carotid (C2) segment penetrating injury which was poorly controlled in spite proximal common carotid vascular clamp occlusion and direct finger pressure in the submandibular region.

As noted by Lee et al., penetrating carotid injuries are significantly more lethal with a 22 % mortality rate, compared with a 7 % mortality rate in the BCI group. In addition, the presence of shock was associated with a higher mortality rate of 41 %, compared with 8 % in the no-shock group [20].

Reva and other authors have cautioned against surgical repair of the distal ICA near the skull base, due to technical difficulty and have discouraged thrombectomy involving the distal portion of ICA due to the risks of thrombus migration. Instead, an endovascular approach for select zone III and zone I injuries is advised [20,21]. This supports the du Toit's recommendations for Zone III ICA injury, as well as the reported technical success of 100 % with a 30-day stroke rate and mortality of 5 %, respectively [22]. These result compares well to du Toit's open surgery results of a mortality of 18 % and a stroke rate of 5 % after arterial repair [3].

The mechanism of injury, shock state, pre-operative GCS as well as the Zone of Injury have a significant bearing on the surgical approach. In view of the clinical presentation of our patient, it was felt that local open control via FBCT and endovascular stent repair would be prudent.

We have done an extensive review of the literature. It appears that our case report is the first to describe the management of a case of a stab wound to the left neck managed by a surgical left neck exploration, FCBT, for initial control of massive hemorrhage and a subsequent interventional radiology (IR) endovascular stent placement for a zone 3 internal carotid artery injury. The authors hope that the information provided may be instructive to our surgical colleagues and improve the outcomes of patients with complex penetrating neck injuries.

## CRediT authorship contribution statement

**Rebekah L. Bjorklund:** Writing – review & editing, Writing – original draft, Visualization. **Luis Fernandez:** Writing – review & editing, Visualization, Supervision, Investigation, Data curation. **Gary Spiegel:** Investigation, Writing – review & editing. **Brittany Wagner:** Writing – review & editing. **Austin Eagleton:** Supervision, Resources, Data curation.

## Declaration of competing interest

Dr. Fernandez is on the Speakers Bureau for Urgo Medical. All other authors have no competing financial or personal interests that would influence the information provided in this case report.
